# Effectiveness of non-pharmaceutical intervention on sperm quality: a systematic review and network meta-analysis

**DOI:** 10.18632/aging.204727

**Published:** 2023-05-17

**Authors:** Zilong Chen, Zhiming Hong, Shengjie Wang, Junfeng Qiu, Quan Wang, Yangling Zeng, Haowei Weng

**Affiliations:** 1 Shenzhen Traditional Chinese Medicine Hospital, Guangdong 518000, China; 2 The Fourth Clinical Medical College of Guangzhou University of Chinese Medicine, Guangdong 518000, China

**Keywords:** male infertility, sperm quality, non-pharmaceutical interventions, effectiveness, network meta-analysis

## Abstract

Infertility affects about 10% of the world’s population and has been recognized by the WHO as a global public health problem. The aim of this network meta-analysis was to investigate the efficacy of non-pharmaceutical interventions on sperm quality. All randomized clinical trials (RCTs) from the PubMed, MEDLINE, Embase, China national knowledge infrastructure (CNKI), Wanfang database, and Cochrane Library databases evaluating the effectiveness of non-pharmaceutical interventions on semen parameters using network meta-analyses. Results of the ω-3 fatty acid, lycopene, acupuncture, and vitamin suggested evident advantages in improving sperm concentration (MD, 9.93 (95% CI, 7.21 to 12.65)), (MD, 8.79 (95% CI, 2.67 to 14.91)), (MD, 5.40 (95% CI, 2.32 to 8.49)) and (MD, 3.82 (95% CI, 0.70 to 6.94) respectively). Acupuncture has a significant advantage over placebo in improving sperm total motility (MD, 17.81 (95% CI, 10.32 to 25.29)), and the effect of lycopene was obviously greater than that of placebo (MD, 19.91 (95% CI, 2.99 to 36.83)). Lycopene, Coenzyme Q10 (CoQ10), acupuncture, ω-3 fatty acid, and vitamin suggested significant advantages in improving sperm forward motility (MD, 8.64 (95% CI, 1.15 to 16.13), MD, 5.28 (95% CI, 2.70 to 7.86), MD, 3.95 (95% CI, 3.23 to 4.67), MD, 3.50 (95% CI, 2.21 to 4.79)) and (MD, 2.38 (95% CI, 0.96 to 3.80) respectively). This review establishes that non-pharmaceutical interventions, particularly acupuncture, exercise, lycopene, ω-3 fatty acids, CoQ10, zinc, vitamins, selenium, carnitine, or foods rich in these supplements, profitably improve sperm quality that may be used to treat male infertility.

## INTRODUCTION

The World Health Organization (WHO) has declared infertility to be the major global public health problem for the past few decades [1]. Infertility is the failure to become pregnant after twelve months or more of proper and timed unprotected intercourse [2, 3]. It is estimated that approximately 10 to 15 percent of people worldwide are affected by infertility, thus making it a global concern [4]. There are approximately 186 million infertility cases worldwide, and more than half of them are male infertility [5]. Recently, increasing studies have highlighted the influences of inflammation of the reproductive tract, irregular lifestyles, and nutrition deficiency [6, 7] during the pathogenesis of male infertility. In this regard, being overweight and other conditions, such as alcohol abuse, metabolic syndrome, smoking, and environment are strongly associated with reduced sperm quality and fertility. Therefore, the sperm quality can affect ejaculate competitiveness and influence siring success [8, 9]. Additionally, it has been reported that about 30–80% of male infertility is deemed to be due in part to the negative impacts of oxidative stress on the sperm [10, 11]. Oxidative stress takes place when reactive oxygen species (ROS) overpower semen’s anti-oxidation defenses, which destroy proteins, DNA, and lipids [10, 12]. Sperm DNA damage caused by oxidative stress can lead to decreased sperm motility, acrosomal membrane damage, sperm fertilization ability, and ultimately led to fertility decline [13–15]. One randomized clinical trial (RCT) study suggested that resistance exercise might modulate male infertility through anti-inflammatory and anti-oxidation mechanisms [16].

Treatment and management of male infertility include pharmaceutical, non-pharmaceutical, and surgical interventions. Currently, the primary clinical treatment of male infertility drugs includes antioxidants, hormones, hexanone theobromine, L-carnitine (LC), and other drugs [17], which have several disadvantages, such as uncontrollable side effects, expensiveness, case dependence, and poor outcomes. In the meantime, non-pharmaceutical interventions such as acupuncture, massage, moxibustion, and scraping have been developed and applied in the management and treatment of male infertility [18]. Non-pharmaceutical therapies, especially lifestyle, can improve sperm quality, motility, and morphology through treatments such as lifestyle changes, and can improve the overall physical fitness of men [19, 20]. Although the aforementioned systematic reviews with or without network meta-analysis analyzed the efficacy and safety of different treatments for male infertility, comparisons between the effects of different non-pharmaceutical treatments have not been performed. Therefore, our study aimed to verify the comparative effectiveness of non-pharmaceutical treatments for male infertility by performing the network meta-analysis.

## METHODS

### Data sources and searches

For the network meta-analysis, RCTs were searched in PubMed, Embase, MEDLINE, China national knowledge infrastructure (CNKI), Wanfang database, and Cochrane Library databases from initiation to 5 April 2023, using relevant free-text terms. Relevant medical subject terms (MeSH) and text terms were contained in the search terms in the network meta-analysis. This strategic search terms were as follows: “infertility, male” (MeSH Terms) OR (“infertility” (All Fields) AND “male” (All Fields)) OR “male infertility” (All Fields) OR (“male” (All Fields) AND “infertility” (All Fields)) AND (“diet” (MeSH Terms) OR “diet” (All Fields) OR (“exercise” (MeSH Terms) OR “exercise” (All Fields) OR “exercises” (All Fields) OR “exercise therapy” (MeSH Terms) OR (“exercise” (All Fields) AND “therapy” (All Fields)) OR “exercise therapy” (All Fields) OR “exercises” (All Fields) OR “exercised” (All Fields) OR “exerciser” (All Fields) OR “exercisers” (All Fields) OR “exercising” (All Fields)) OR (“acupunctural” (All Fields) OR “acupuncture” (MeSH Terms) OR “acupuncture” (All Fields) OR “acupuncture therapy” (MeSH Terms) OR (“acupuncture” (All Fields) AND “therapy” (All Fields)) OR “acupuncture therapy” (All Fields) OR “acupuncture s” (All Fields) OR “acupunctured” (All Fields) OR “acupunctures” (All Fields) OR “acupuncturing” (All Fields)) OR (“psychotherapies” (All Fields) OR “psychotherapy” (MeSH Terms) OR “psychotherapy” (All Fields) OR “psychotherapies” (All Fields) OR “psychotherapy s” (All Fields)). We then screened a list of references to all the acquired articles including related reviews.

### Study selection

After deduplication, each study is assessed for primary qualifications in the title and abstract, and these articles were screened by two independent researchers (ZMH and SJW). Studies published only as summaries without extra data were excluded. In the case of file screening and data extraction as described above, any discrepancies will be solved by submitting them to the third researcher (ZHC). In accordance with the PRISMA guidelines, the screening process for included studies will be shown in a flow chart [21].

### Data extraction and quality assessment

All data from the included literature were extracted by two independent researchers using Excel software in the same predetermined table. Any differences will be solved by the third reviewer. The extracted data items are as follows: the first author and published year, age, sample size, intervention, daily dosage, treatment time, and some other outcomes of interest. The primary outcome was the pregnancy rate. Secondary outcomes included sperm parameters, e.g., sperm concentration, sperm total motility, sperm forward motility, sperm quality, and sperm count. For the results of sperm parameters, our study extracted the relevant metrics applied to evaluate the inclusion study. These studies included both fresh and cryopreserved semen. For these fresh and cryopreserved samples, the control groups for each study had the same sample collection conditions as the intervention groups. There are, therefore, minor effects on the conclusions.

### Risk-of-bias assessment

The risk of bias (ROB) for the inclusion study was confirmed for each result by two independent reviewers. The ROB for included studies was evaluated by the Cochrane ROB tool [22]. Differences will be solved by the third reviewer. For RCTs, each ROB project was classified as “low risk” if bias was suspected to significantly alter these results; if the expectation bias caused some uncertainty in the outcomes, it would be “unclear”; alternatively, it would be “high risk” if the expected bias would completely change the outcome. A funnel plot adjusted for comparison was drawn to test any main publication bias in this study [23].

### Data synthesis and analysis

This study performed a network meta-analysis (NMA) using STATA 14. The comparative effect of all interventions was assessed by computing the mean difference by applying the random effects model. The estimated significance of the resulting *p*-value was set at < 0.05 with a 95% confidence interval (CI). The subgroup analyses of clinical results for diverse interventions were performed to elucidate heterogeneity between studies. Moreover, our study conducted a network diagram of the result; the node size represented the number of patients randomly assigned to each intervention; the nodal line thickness corresponded to the number of researchers evaluating each comparison [24]. The ranking probability of each intervening measure in each possible ranking was assessed. Our study concluded the intervention hierarchy and reported that it is the surface under the cumulative ranking curve (SUCRA); the intervention with a SUCRA value of 100 is definitely the best, and the intervention with a value of 0 is definitely the worst [23].

## RESULTS

### Study characteristics

**Figure 1:**
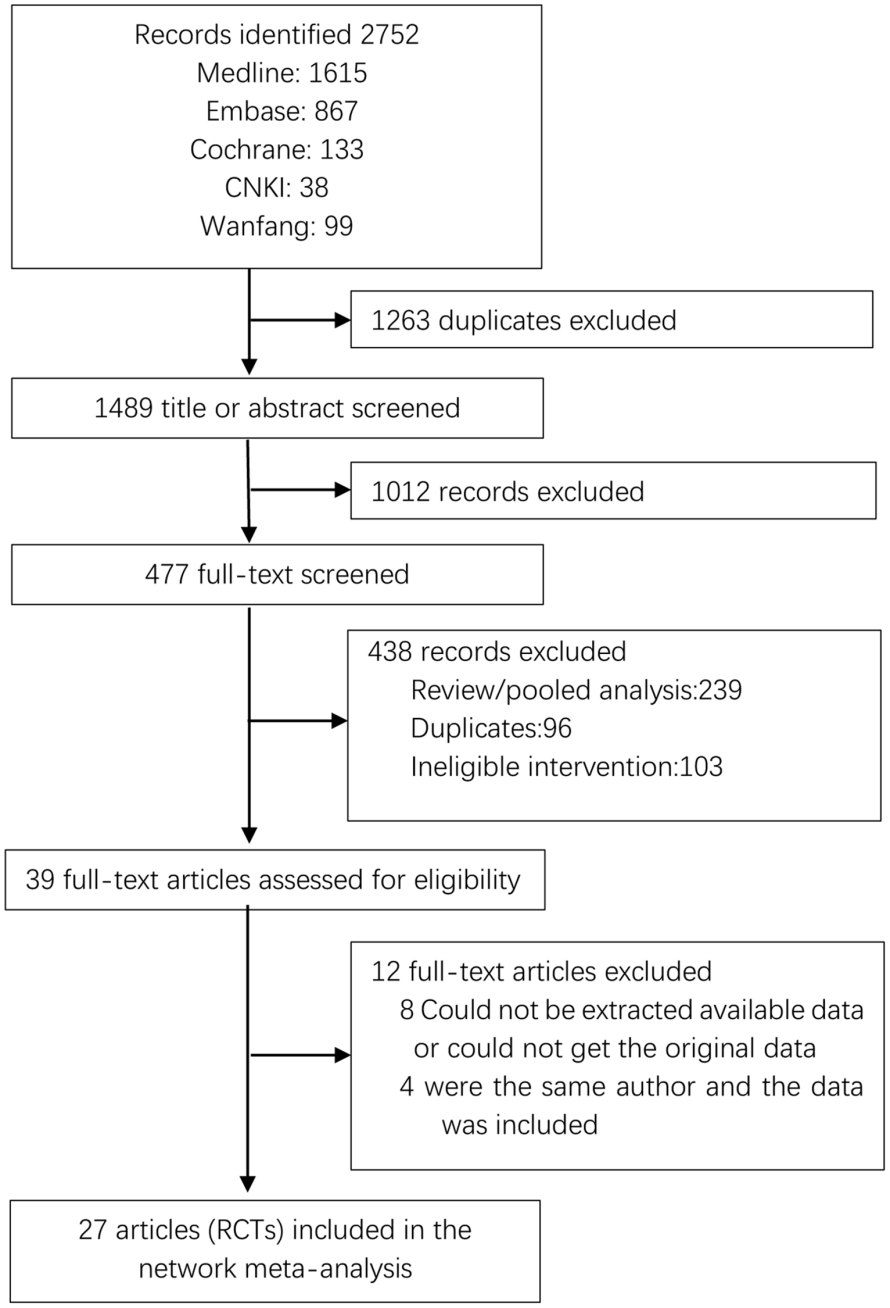
**PRISMA flow diagram showing study search and selection.** Abbreviation: RCTs: randomized controlled trials.

**Figure 2:**
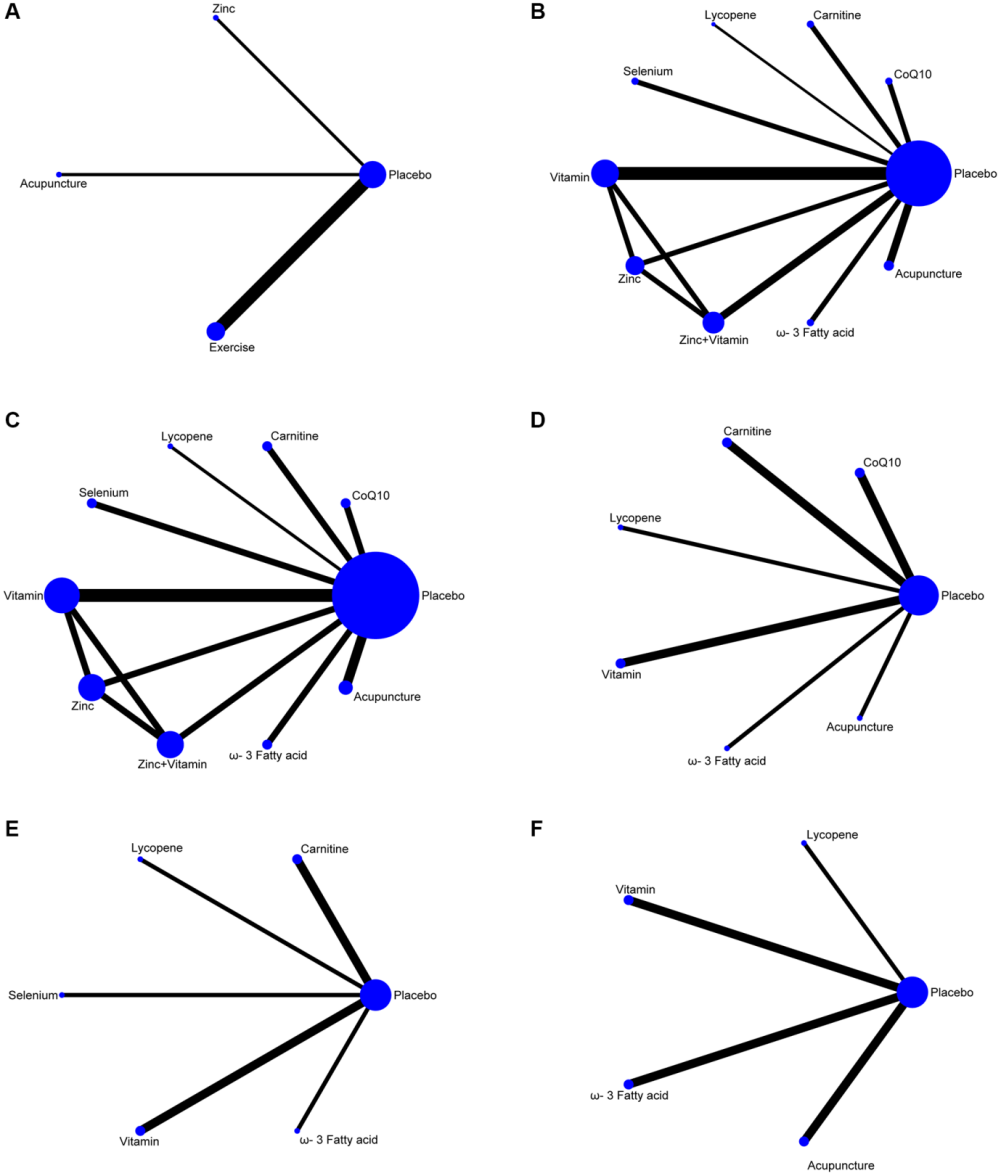
**Network plots of non-pharmaceutical interventions on sperm parameters.** (**A**) Pregnancy rate. (**B**) Sperm concentration. (**C**) Sperm total motility. (**D**) Sperm forward motility. (**E**) Sperm quality. (**F**) Sperm count.

The screening process for the included studies is indicated in Figure 1. A total of 2508 records were screened, and 27 RCTs (*n* = 4008 patients) were included in this analysis [25–47]. The network plots of pregnancy rate, sperm concentration, sperm total motility, sperm forward motility, sperm quality, and sperm count analysis are indicated in Figure 2. In the analysis of primary outcomes disaggregated by intervention, there were 11 interventions: CoQ10, zinc, ω-3 fatty acid, carnitine, selenium, lycopene, vitamin, zinc + vitamin, exercise, acupuncture, and placebo. Exercise, acupuncture, and vitamins were the most commonly investigated interventions in all comparisons (4 trials, respectively). With regard to acupuncture, the included studies were divided into two categories: acupuncture (2 trials) and electroacupuncture (2 trials). In addition, the most frequent interventions were CoQ10 (3 trials), selenium (3 trials), and zinc + vitamin (3 trials).

**Figure 3:**
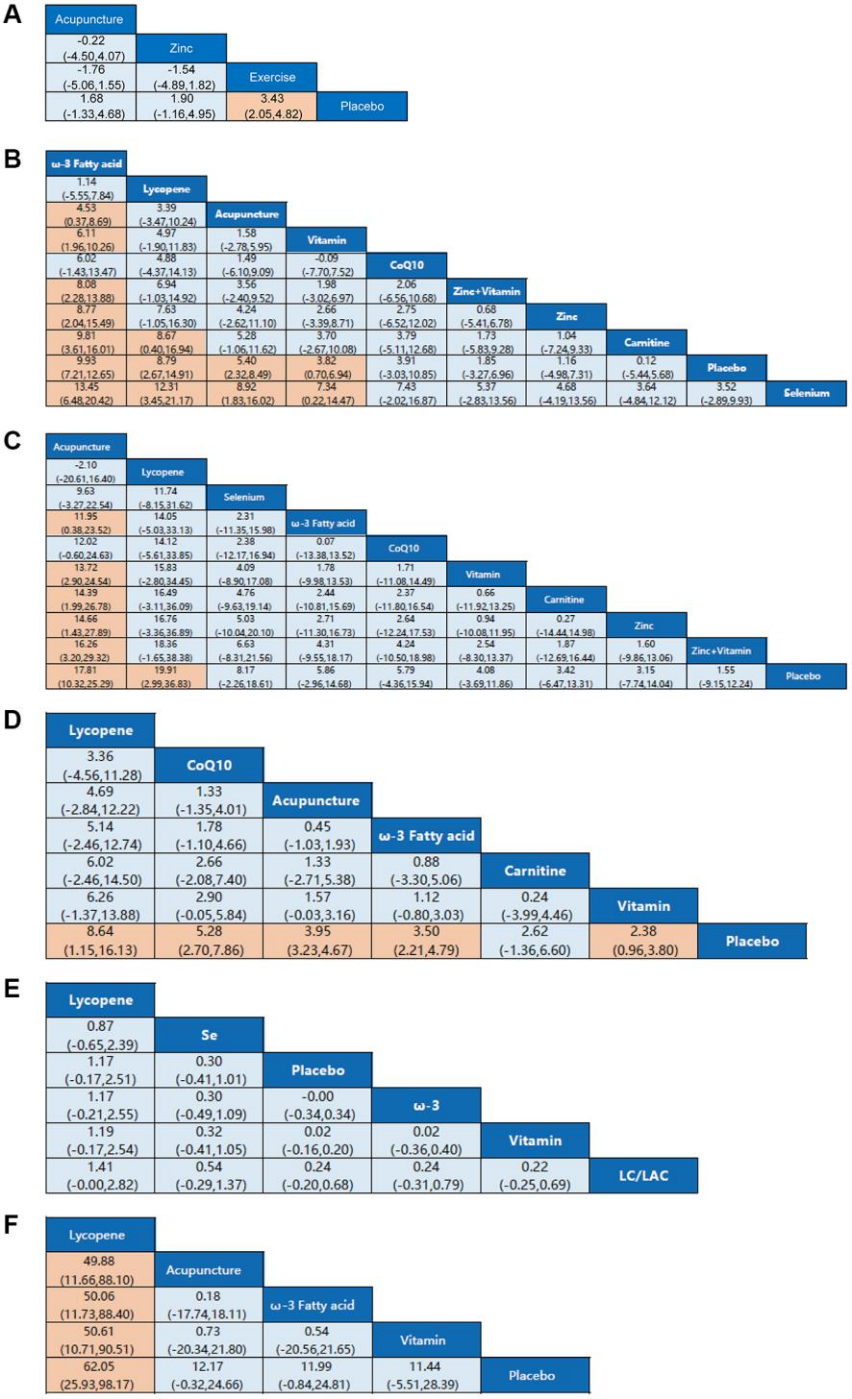
**Network meta-analysis estimates for efficacy of non-pharmaceutical interventions on sperm parameters.** (**A**) Pregnancy rate. (**B**) Sperm concentration. (**C**) Sperm total motility. (**D**) Sperm forward motility. (**E**) Sperm quality. (**F**) Sperm count. Non-pharmaceutical interventions are listed in order of efficacy ranking according to SUCRAs. Comparisons should be read from left to right. Statistically significant differences are shown in orange.

The baseline and critical features of the included studies are indicated in Table 1. Twenty-two of the included studies lasted longer than three months. The average number of people included in the study was 148 individuals, ranging from 8 to 521 patients. In all trials, patients were aged 18–45 years. Eleven (40.7%) trials recruited patients from Europe, three (11.1%) from Asia, one (3.7%) from North America, one (3.7%) from Australia, one (3.7%) from South America, and one (3.7%) from South Africa. In the rest of the trials, nine (33.3%) proceeded in the Middle East. Figure 3 SUCRA ranking of the interventions based on their treatment effect and cumulative probability plot indicates that all interventions can treat male infertility compared with placebo. The funnel plots of these comparisons show evidence of fundamental symmetry.

**Table 1:** Summary of the RCT studies investigating the effect of non-pharmaceutical interventions on sperm parameters.

**Study**	**Country**	**Age**	**Sample size**	**Intervention**		**Daily dosage**	**Treatment time**	**Outcome**	**References**
				**Intervention**	**Intervention**				
Ketabchi et al. 2018	Iran	33.4 (7.6)/32 (6.5)	51	Acupuncture	Mock acupuncture	NA	180d	Improved sperm quality	[27]
Sun et al. 2016	China	32 (3)/31 (3)	100	Acupuncture	Mock acupuncture	40 min daily	70d	Improved sperm density, motility and viability	[42]
Jin et al. 2017	China	32.68 (0.95)/30.33 (0.91)	72	Acupuncture	Mock acupuncture	30 min daily	60d	Increasing sperm motility and vitality	[34]
Yu et al. 2019	China	31.45 (0.84)/29.52 (0.81)	121	Acupuncture	Mock acupuncture	30 min daily	60d	Increasing sperm motility and count	[45]
Nadjarzadeh et al. 2012	Iran	34.17 (4.52)/34.67 (6.69)	47	CoQ10	Placebo	200 mg/d	90d	Improved sperm functions	[25]
Balercia et al. 2009	Italy	27–39	57	CoQ10	Placebo	200 mg/d	180d	Improved sperm kinetic features	[32]
Safarinejad et al. 2012	Iran	31/32	228	CoQ10	Placebo	200 mg/d	182d	Improved sperm density, motility and morphology	[38]
Lenzi et al. 2004	Italy	20–40	56	Carnitine	Placebo	3 g/d	180d	Increasing sperm motility	[28]
Balercia et al. 2005	Italy	20–40	59	Carnitine	Placebo	3 g/d	180d	Improved sperm kinetic features	[47]
Rolf et al. 1999	Germany	NA	31	Vitamin	Placebo	1000 mg/800 mg/d	56d	Improved sperm concentration, motility and morphology	[29]
Kessopoulou et al. 1995	UK	26–49/25–37	30	Vitamin	Placebo	600 mg/d	712d	Increasing sperm motility	[30]
Greco et al. 2005	France	NA	64	Vitamin	Placebo	1000 mg/d	60d	Improved sperm concentration and motility	[31]
Haghighian et al. 2015	Iran	32.98 (5.35)/34.12 (4.79)	54	Vitamin	Placebo	600 mg/d	84d	Improved sperm motility	[33]
Omu et al.1997	Kuwait	37.8 (7.9)/38.12 (8.2)	97	Zinc	Placebo	500 mg/d	90d	Improved sperm quality and count	[26]
Ebisch et al. 2005	Netherlands	32.3–37.0/31.0–38.0	40	Zinc + Vitamin	Placebo	71 mg/d	182d	Improved sperm concentration	[46]
Wong et al. 2002	South Africa	34.4 (4.7)32.9 (4.6)	211	Zinc + Vitamin	Placebo	71 mg/d	182d	Increasing sperm variables	[43]
Raigani et al. 2013	Iran	NA	83	Zinc + Vitamin	Placebo	270 mg/d	112d	No differences	[39]
Martínez-Soto et al. 2016	Spain	35 (0.8)/35.6 (1.0)	74	ω-3 Fatty acid	Placebo	1500 mg/d	70d	No differences	[35]
Safarinejad et al. 2009	Iran	32 (9)/32 (10)	211	ω-3 Fatty acid	Placebo	1.84 g/d	224d	Improved sperm count and motility	[37]
Safarinejad et al. 2008	Iran	31 (9)/31 (9)	468	Selenium	Placebo	200 ug/d	182d	Improved sperm quality	[36]
Scott et al. 1998	UK	32.6 (1.1)/32.9 (1.5)	34	Selenium	Placebo	100 ug/d	90d	Improved sperm motility	[41]
Hawkes et al. 2009	California	18–45	42	Selenium	Placebo	300 mg/d	336d	No differences	[44]
Nouri et al. 2019	Iran	32.89 (2.33)/32.15 (2.16)	44	Lycopene	Placebo	25 mg/d	84d	Improved sperm count and concentration	[40]
Behzad et al. 2017	Germany	25–40	386	Exercise	No-exercise	/	168d	Increasing pregnancy rate	[68]
Behzad et al. 2017	Germany	25–40	521	Exercise	No-exercise	/	168d	Increasing pregnancy rate	[69]
Behzad et al. 2018	Germany	25–40	407	Exercise	No-exercise	/	168d	Increasing pregnancy rate	[16]
Behzad et al. 2020	Germany	25–40	420	Exercise	No-exercise	/	252d	Increasing pregnancy rate	[70]

### Network meta-analysis

To assess the influence of non-pharmaceutical treatments on sperm quality, twenty-six studies were included. Usage of CoQ10, zinc, omega-3 fatty acids, carnitine, selenium, lycopene, vitamin, zinc + vitamin, exercise, and acupuncture are relatively frequent in these RCTs. The number of RCTs and the homogeneity between them make our network meta-analysis suitable to test the effectiveness of the various non-pharmaceutical interventions on semen parameters, such as pregnancy rate, sperm concentration, sperm total motility, sperm forward motility, sperm quality, and sperm count. The network diagrams demonstrate the correlation of all available evidence. The line thickness represents the number of included studies, and the node size indicates the sample size (Figure 2).

#### Pregnancy rate

Four studies resulted in the network meta-analysis for the non-pharmaceutical interventions on pregnancy rate (Figure 2A). The SUCRA rankings suggest that the top three interventions are exercise, zinc, and acupuncture (Table 2). The network meta-analyses outcomes are indicated in Figure 3A. Compared with placebo, acupuncture indicated significant advantages in treating pregnancy rate (MD, 1.68 (95% CI, −1.33 to 4.68)). Zinc was obviously more effective than the placebo (MD, 1.90 (95% CI, −1.16 to 4.99)). Exercise had better efficacy than the placebo (MD, 3.43 (95% CI, 2.05 to 4.82)). We found no detectable differences in any of the other comparisons (Figure 3A).

**Table 2:** SUCRA ranking probabilities of different treatments.

	**Treatment**	**SUCRA**	**Rank**		**Treatment**	**SUCRA**	**Rank**
Pregnancy rate	Exercise	88.7	1	Sperm total motility	Acupuncture	91.6	1
	Zinc	53.3	2		Lycopene	89.8	2
	Acupuncture	49.9	3		Selenium	60.8	3
	Placebo	8.2	4		ω-3 Fatty acid	50	4
Sperm concentration	ω-3 Fatty acid	95	1		CoQ10	48.8	5
	Lycopene	87.2	2		Vitamin	41.4	6
	Acupuncture	70.1	3		Carnitine	37.4	7
	Vitamin	57.9	4		Zinc	36.8	8
	CoQ10	56.5	5		Zinc + Vitamin	27.8	9
	Zinc + Vitamin	40.7	6		Placebo	15.5	10
	Zinc	34.8	7	Sperm quality	Lycopene	94.3	1
	Carnitine	27.2	8		Selenium	68.3	2
	Placebo	23.1	9		Placebo	43.6	3
	Selenium	7.5	10		ω-3 Fatty acid	42.1	4
Sperm forward motility	Lycopene	91.1	1		Vitamin	38.9	5
	CoQ10	79.5	2		Carnitine	12.7	6
	Acupuncture	62.1	3	Sperm count	Lycopene	99.6	1
	ω-3 Fatty acid	50.2	4		Acupuncture	50.2	2
	Carnitine	37.1	5		ω-3 Fatty acid	49.5	3
	Vitamin	28.2	6		Vitamin	47	4
	Placebo	1.9	7		Placebo	3.7	5

Abbreviation: SUCRA: surface under the cumulative ranking curve.

#### Sperm concentration

Twenty studies resulted in the network meta-analysis for the non-pharmaceutical interventions on sperm concentration (Figure 2B). The top four ranked interventions were ω-3 fatty acid, lycopene, acupuncture, and vitamin, as the SUCRA rank indicated (Table 2). The outcomes of the network meta-analyses are shown in Figure 3B. The ω-3 fatty acid, lycopene, acupuncture, and vitamin suggested evident advantages in treating the sperm concentration compared with placebo (MD, 9.93 (95% CI, 7.21 to 12.65)), (MD, 8.79 (95% CI, 2.67 to 14.91)), (MD, 5.40 (95% CI, 2.32 to 8.49)) and (MD, 3.82 (95% CI, 0.70 to 6.94)). ω-3 fatty acid, lycopene, acupuncture, and vitamin were statistically significantly better than the selenium (MD, 13.45 (95% CI, 6.48 to 20.42)), (MD, 12.31 (95% CI, 3.45 to 21.17)), (MD, 8.92 (95% CI, 1.83 to 16.02)) and (MD, 7.34 (95% CI, 0.22 to 14.47)). The ω-3 fatty acid and lycopene had significantly greater effects than carnitine (MD, 9.81 (95% CI, 3.61 to 16.01)) and (MD, 8.67 (95% CI, 0.40 to 16.94)). The ω-3 fatty acid was also statistically significantly better than acupuncture, vitamin, zinc + vitamin, and zinc. In addition, we found no detectable differences in any of the other comparisons (Figure 3B).

#### Sperm total motility

Sixteen studies resulted in the network meta-analysis for the non-pharmaceutical interventions on sperm total motility (Figure 2C). As the SUCRA rankings show, the top two interventions are acupuncture and lycopene (Table 2). The results are indicated in Figure 3C. Acupuncture has a significant advantage over placebo in treating sperm total motility (MD, 17.81 (95% CI, 10.32 to 25.29)). The effect of lycopene was obviously greater than that of placebo (MD, 19.91 (95% CI, 2.99 to 36.83)). The acupuncture was also statistically significantly better than ω-3 fatty acid (MD, 11.95 (95% CI, 0.38 to 23.52)), vitamin (MD, 13.72 (95% CI, 2.90 to 24.54)), carnitine (MD, 14.39 (95% CI, 1.99 to 26.78)), zinc (MD, 14.66 (95% CI, 1.43 to 27.89)) and zinc+vitamin (MD, 16.26 (95% CI, 3.20 to 29.32)). We found no detectable differences in any of the other comparisons (Figure 3C).

#### Sperm forward motility

Nine studies resulted in the network meta-analysis for the non-pharmaceutical interventions on sperm forward motility (Figure 2D). The SUCRA rankings suggest that the top three interventions are lycopene, CoQ10 and acupuncture (Table 2). The network meta-analyses outcomes are suggested in Figure 3D. Compared with placebo, lycopene suggested significant advantages in treating sperm forward motility (MD, 8.64 (95% CI, 1.15 to 16.13)). CoQ10 was obviously more effective than the placebo (MD, 5.28 (95% CI, 2.70 to 7.86)). Acupuncture had a better efficacy than the placebo (MD, 3.95 (95% CI, 3.23 to 4.67)). ω-3 fatty acid and vitamin were also statistically obviously better than placebo (MD, 3.50 (95% CI, 2.21 to 4.79)) and (MD, 2.38 (95% CI, 0.96 to 3.80)). We found no detectable differences in any of the other comparisons (Figure 3D).

#### Sperm quality

Seven resulted in the network meta-analysis for the non-pharmaceutical interventions on sperm quality (Figure 2E). The top two ranked interventions were lycopene and selenium, as the SUCRA rank indicated (Table 2). The main outcomes are indicated in Figure 3E. Lycopene, selenium, ω-3 fatty acid, vitamin, and carnitine were not also statistically obviously better than the placebo. We found no detectable differences in any of the other comparisons (Figure 3E).

#### Sperm count

Seven resulted in the network meta-analysis for the non-pharmaceutical interventions on sperm count (Figure 2F). The number one intervention is lycopene, as indicated in the SUCRA rankings (Table 2). The results are indicated in Figure 3F. Lycopene had a better efficacy than acupuncture (MD, 49.88 (95% CI, 11.66 to 88.10)), ω-3 fatty acid (MD, 50.06 (95% CI, 11.73 to 88.40)), vitamin (MD, 50.61 (95% CI, 10.71 to 90.51)) or placebo (MD, 62.05 (95% CI, 25.93 to 98.17)). Besides, we found no detectable differences in any of the other comparisons (Figure 3F).

### Quality assessment and publication bias

**Figure 4:**
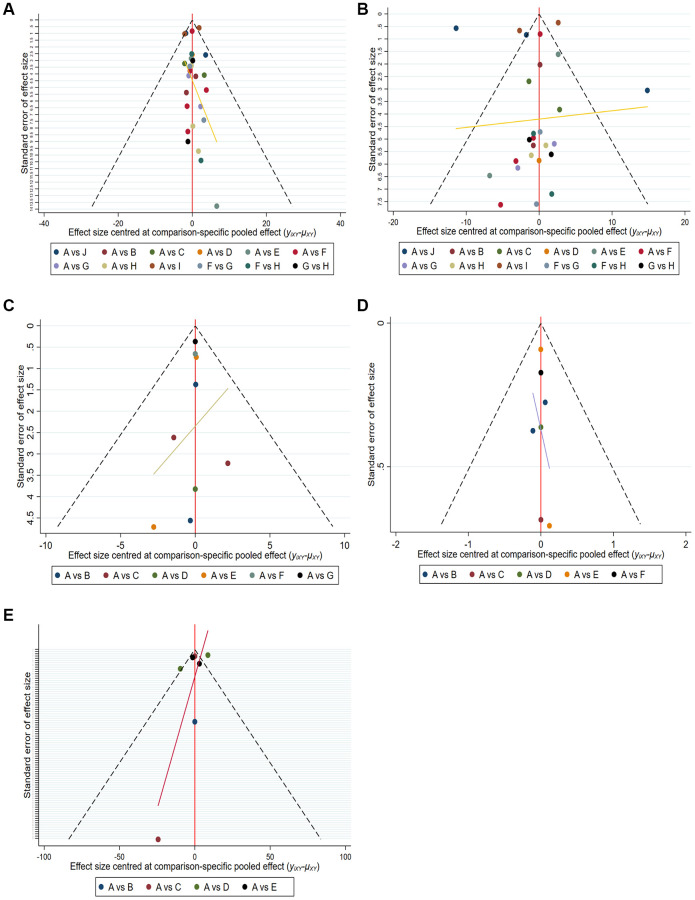
**Comparison-adjusted funnel plot for mean overall change in sperm parameters in all comparisons.** (**A**) Pregnancy rate. (**B**) Sperm concentration. (**C**) Sperm total motility. (**D**) Sperm forward motility. (**E**) Sperm quality.

Twenty-seven articles were included in the network meta-analysis based on inclusion and exclusion criteria. Funnel plots, Begg’s test and Egger’s test were used to assess the quality and publication bias of the included studies. The funnel plot of logarithmic WMD in the included studies was symmetric, indicating no significant publication bias (Figure 4).

## DISCUSSION

**Figure 5:**
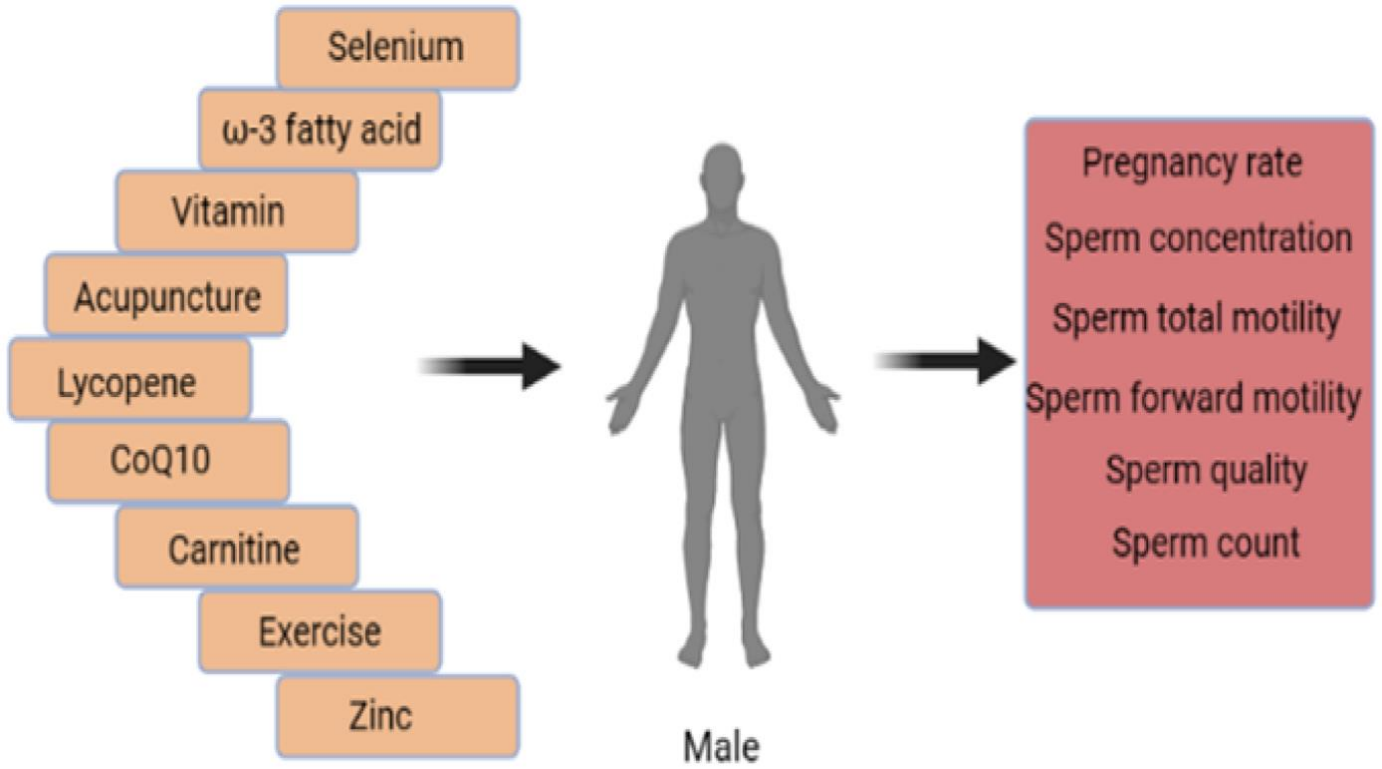
All interventions were able to improve male infertility.

This RCTs network meta-analysis provides the most extensive analysis to date of the influences of non-pharmaceutical interventions on sperm parameters, conducted worldwide, in different healthcare settings, and under different experimental practices (Figure 5). The network meta-analysis indicates the beneficial effects of exercise, zinc, and acupuncture on pregnancy rates. Acupuncture and supplementations with omega-3 fatty acids, lycopene, and vitamins had significant beneficial effects on sperm concentration. Acupuncture and lycopene supplementation also had significant beneficial effects on total sperm motility. Acupuncture and supplementation with lycopene or CoQ10 had significant beneficial effects on sperm forward motility. Lycopene supplementation significantly improved sperm count. The network meta-analysis also indicated that some other dietary supplements might help improve male fertility.

The ultimate goal of male infertility treatment is to improve semen quality and eventually increase pregnancy rates. These RCTs concluded that these non-pharmaceutical interventions were effective in improving several measures of male fertility, including semen quality parameters as well as pregnancy and live birth rates [48, 49]. However, all of these showed positive results for reproductive function and were significant from a fertility perspective. Diet, exercise, and acupuncture have been associated with increased fertility in pregnant women [48, 50]. It has also been associated with increased endometrial thickness, embryo implantation, and live birth in women undergoing *in vitro* fertilization [49, 51]. The results of these studies are consistent with our results, which further confirmed that exercise, zinc, and acupuncture are positively associated with increased clinical pregnancy rates.

Assessing the fertilizing potential of an ejaculate generally includes tests of sperm function, as well as evaluation of sperm morphology, motility profiles, concentration, viability ability to acrosome-react and to penetrate oocytes [52]. Thus, assessment of sperm quality in a semen sample is the only and standard method to disclose its potential fertility [53]. Omega-3 fatty acids have antioxidant and anti-inflammatory properties that may alter the function of cell membranes through the integration into cell membrane [54], which may explain this mechanism by which Omega-3 fatty acids affect spermatogenesis. Meanwhile, successful sperm fertilization has been shown to depend on the lipid composition of the sperm membrane [55]. Consistent with these findings, this current RCTs meta-analysis indicates a positive impact of Omega-3 fatty acid supplementation on sperm concentration. Nevertheless, other RCTs with large participant samples are needed to confirm the beneficial influences of omega-3 supplementation on sperm parameters. Lycopene, one of the most powerful antioxidants, is an aliphatic hydrocarbon with antioxidant defense against lipid peroxidation [56], which is reported to be present in high concentrations in male testes. It may play an important role in spermatogenesis as an important substance [57]. It was reported to affect several biological processes in prostate disease. In addition, it is also shown to prevent structural and functional injury to the testes and changes in sperm quality caused by oxidative stress [58].

As a traditional treatment, acupuncture may be an underlying treatment option for asthenospermia. Recent studies have shown that acupuncture is effective in treating most kinds of male infertility [59, 60]. The main acupoints selected include Guanyuan, Qihai, Zusanli, Sanyinjiao and Baihui. Guanyuan is where the vitality is, filling the strong true Qi and strong congenital. Guanyuan and Qihai are closely related to reproduction. Taking Sanyinjiao to regulate and replenish the three Yin meridians is a common acupoint for invigorating the spleen and removing dampness. It has the function of nourishing Yin and blood, activating blood circulation, and unblocking collaterals. Zusanli and Yinlingquan have the effect of tonifying the spleen and stomach, regulating Qi and blood, cultivating the acquired essence, and nourishing the congenital kidney Qi. The Bai hui belongs to the Dumai, which is the meeting of all Yang. It can replenish Qi and promote Yang. It is compatible with other acupoints to harmonize Yin and Yang. When all acupoints are used together, the kidney Yang can be strengthened, the kidney Yin can be cultivated, the kidney essence can be enriched, and the innate and acquired complement can promote the growth of sperm and enhance the vitality of sperm. At the same time, the essence can be vigorous and strong, and sometimes, the two essences can fight together, which guarantees pregnancy. A Norwegian study showed that about 20 percent of infertile males prefer alternative treatments such as acupuncture [61]. Summary data on sperm concentration and motility indicated that acupuncture could be used in patients with oligozoospermia and asthenospermia. A variety of mechanisms could elucidate the effectiveness of acupuncture. Siterman et al. [62] showed that acupuncture may decrease lipid peroxidation in sperm or genital inflammation by increasing immune responses. Therefore, our results are consistent with the above previous studies.

CoQ10 is an antioxidant that plays a key effect on the electron transport systems. As Balercia and Safarinejad et al. pointed out, CoQ10 suppresses the formation of organic peroxides in the semen and thus may decrease the oxidative stress of sperm cells [32, 63]. This is consistent with our research results, CoQ10 may improve sperm quality and sperm motility through antioxidants. Over the past few years, interest in the molecule has grown as a supplement to treat fertility. In this study, sperm parameters showed an overall improvement. All content types of carnitines, e.g., LC, L-acetylcarnitine (LAC), or a combination of both carnitines (LAC and LC) had all been demonstrated to augment sperm motility. This study indicated that carnitine supplementation also had certain profitable functions on sperm concentration and sperm total motility. Furthermore, carnitine is participated in the transport of long-chain fatty acids to the mitochondrial matrix for the β-oxidation and exerts antioxidative activity by enhancing the antioxidant enzyme expression [47]. Research data suggest that carnitine therapy is effective in ameliorating fertilization ability and sperm function. The primary antioxidants measured as supplements with beneficial effects on sperm quality parameters were zinc, selenium, and vitamins. On one side, selenium is crucial for spermatogenesis and plays a key function in augmenting the expression and activity of glutathione peroxidase-1 [64]. On the other side, zinc is also an antioxidant that has membrane stabilizing activity through inhibition of membrane-bound oxidases [65]. In the end, in a cross-sectional study, higher vitamin C and E intake were associated with higher sperm count and motility [66]. Our network meta-analysis also indicated that zinc, selenium, and vitamin supplementation may observably add to the sperm quality of infertile males.

According to the fifth edition of the World Health Organization's semen quality analysis standards, the normal reference value of sperm survival rate is above 58%, and the probability of pregnancy for women will be relatively high [67]. Whether or not to conceive normally is also related to various factors such as sperm count, concentration, motility, morphology, and quality of seminal plasma [8]. Of course, the higher the amount of semen, the better the quality of the sperm, and the higher the success rate of pregnancy.

### Limitations

However, there are some limitations to the network meta-analysis conducted. Firstly, some non-English works of literature were not reviewed because they could not be translated into common languages. Still, combined data from non-English studies could change current analyses of male infertility. Secondly, a few studies involve different subgroups, making it difficult to conduct an overall comparison. Moreover, the elimination of low-quality studies and subgroup analyses from the network meta-analysis addressed this problem to some extent, but this remained a limitation of this study. Third, the results of this study show that non-pharmaceutical interventions can improve sperm quality, and further research is needed on the direct effect of non-pharmaceutical interventions on fertility. Fourth, although sperm quality is an important indicator of the pregnancy rate, there are many factors that affect the pregnancy rate. Therefore, the pregnancy rate cannot be used as the most important index and gold standard to judge the clinical effect of male infertility. Fifth, the reference standard of semen detection varies from research unit to research unit, so further standardized research is needed. Finally, due to the variety of male infertility types and interventions, non-pharmaceutical interventions were selected for all interventions to be further classified. In addition to all the limitations, our network meta-analysis evaluated and established studies comparing all non-pharmaceutical interventions in male infertility, which is an important aspect of this work.

## CONCLUSION

The study conducts the most extensive analysis to date of the influences of non-pharmaceutical interventions, such as acupuncture, exercise, food, supplements, and nutrients on sperm quality parameters. This study summarizes that the dietary supplementation with some antioxidants, notably selenium, zinc, omega-3 fatty acids, CoQ10, carnitine, acupuncture, exercise, and some foods rich in these supplements can profitably regulate pregnancy rate and sperm quality parameters and impact male fertility. The small number of included studies on supplements and acupuncture resulted in a small sample size for inclusion, and the high heterogeneity of the findings, meaning that further research may lead to changes in the effect estimates outlined in the meta-analysis. Therefore, more RCTs with larger samples size are needed to verify how these supplements influence sperm parameters and fertility.
